# Risks to the clinician of risk management: recalled and anticipated consequences of decision-making

**DOI:** 10.3389/fpsyt.2025.1484372

**Published:** 2025-02-27

**Authors:** Alexander Challinor, Sahil Bhandari, Sean Boyle, Mark Gabbay, Pete Wilson, Pooja Saini, Rajan Nathan

**Affiliations:** ^1^ Department of Primary Care and Mental Health, University of Liverpool, Liverpool, United Kingdom; ^2^ Department of Research and InnovationMersey Care NHS Foundation Trust, Liverpool, United Kingdom; ^3^ Cheshire and Wirral Partnership NHS Foundation Trust, Chester, United Kingdom; ^4^ National Institute for Health Research Applied Research Collaboration, Liverpool, United Kingdom; ^5^ Health Education North West, Manchester, United Kingdom; ^6^ School of Psychology, Liverpool John Moores University, Liverpool, United Kingdom; ^7^ School of Medicine, University of Chester, Chester, England, United Kingdom

**Keywords:** mental health, healthcare services, psychiatric care, mental health care, inpatient, decision-making, fear

## Abstract

**Background:**

Despite extensive literature studying how we make decisions in the face of uncertainty, the empirical study of real-world clinical decision-making in mental health practice remains limited. Decisions in clinical settings are not just made on the basis of clinical factors. A key non-clinical influence on decision making is the clinician’s concerns about the ‘threat’ to themselves from a future adverse incident and the subsequent retrospective scrutiny of their decision-making. A better understanding of non-clinical processes is essential to inform better ways of guiding effective decision-making. More specifically, delineating the nature of this ‘threat’ process will also inform approaches to patient safety.

**Aims:**

The objective of the current study was to delineate consequences recalled and anticipated by mental health clinicians making decisions under uncertainty.

**Methods:**

This was an analysis of data arising from six focus group discussions with professionals involved in decisions to admit patients to psychiatric hospitals (consultant psychiatrists, approved mental health practitioners, crisis resolution home treatment teams, and liaison psychiatry practitioners) in one National Health Service Trust, UK. The data were thematically analyzed to identify the nature of ‘threat’ processes that arise in clinical decision-making.

**Results:**

Themes identified included (i) the location of the effect of the anticipated/recalled consequence(s), (ii) the location of the origin of the consequence, and (iii) the nature of the consequence. The recalled and anticipated consequences of decision-making were overwhelmingly, but not exclusively, negative. The consequences were largely perceived to be directed towards the self (i.e., the clinician) and were considered to originate from external scrutiny by peers, organizational leadership, and the patient safety system/processes.

**Conclusions:**

The process of making decisions to admit patients to hospital consistently involved the decision-maker’s concern with the future consequences for them, either from a prior or future adverse event. The findings of this study, alongside other evidence of the complexity of decision-making, have implications for improving and studying clinical decision-making (and, by extension, patient care and outcomes), patient safety responses, and professional well-being.

## Introduction

Mental health service delivery encompasses individuals having to face decisions that involve significant complexity and uncertainty. Despite extensive empirically based literature studying how we make decisions in the face of uncertainty, investigations into real-world clinical decision-making in mental health practice remains limited (1, 2). The complex decisions within risk assessment management involve the trade-offs of unknown imperfect options, uncertain outcomes, an inadequate understanding of our patient’s wishes, and a mixture of objective data and subjective judgements (3). This clinical decision-making process recruits complex cognitive processes where clinical decision-making in everyday practice needs to be investigated to consider the wide range of factors that professionals encounter. Current models of healthcare quality recommend that all decisions are evidence-based and patient-centered. Research assessing decision-making in mental health has not begun by studying the foundations of decision theory. They have rather focused on *what* decisions clinicians make (i.e., the probabilistic outcomes) rather than *how* they make decisions (i.e., the process) (4). The studies that have looked at how decisions are made have done so with a greater focus on patient or disorder-based factors (2, 5–7).

Research has focused on examining ways in which decision-making may deviate from what may be predicted, representing systematic patterns of cognitive biases (8, 9). These phenomena can potentially have enormous implications for health care decision-making. One decision within the scope of risk management is the decision to admit a patient to an inpatient mental health service. In the National Health Service (NHS), United Kingdom (UK), this decision can involve a variety of mental health professionals and services. For many patients with acute psychiatric problems, their first option in a crisis is the accident and emergency department. The emergency department setting environment is ill-suited to mental healthcare delivery. Conducting brief crisis assessment outside the patient’s usual social context can influence a clinician’s decision to admit to a psychiatric hospital (10). Alternatively, the emergency assessment may be undertaken by the crisis resolution and home treatment teams (CRHTT), a team at the interface between acute and community mental health, managing individuals that are often placed in difficult acute scenarios (11, 12). CRHTT are multi-disciplinary in nature with front-line assessments conducted by team members with different backgrounds, roles and experiences (12). However, in the NHS, the decision to admit an individual to hospital will usually involve a principal decision-maker, the psychiatrist, particularly if the decision is complex or if there is consideration of involuntary detention (2, 13).

**Figure 1:**
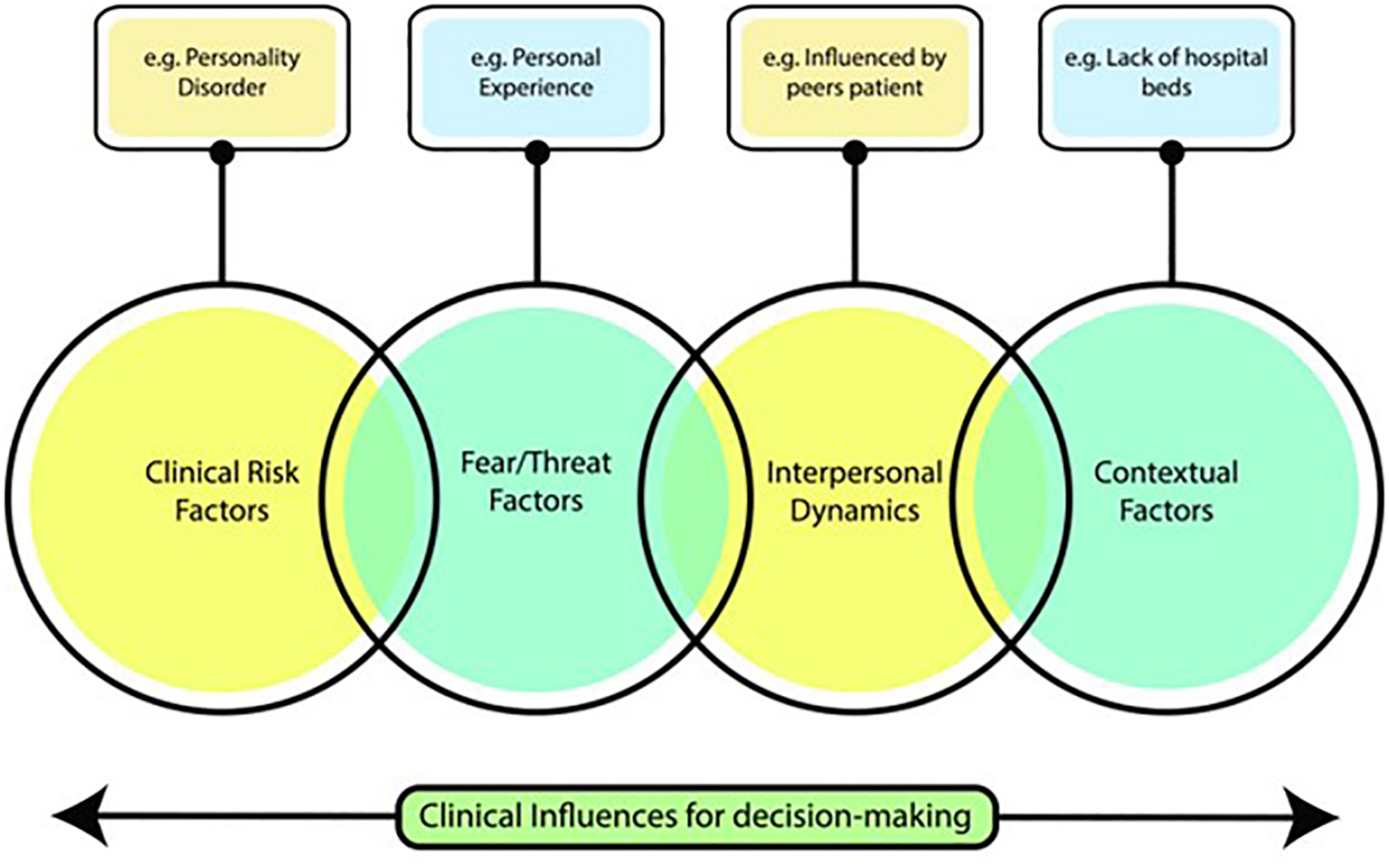
Representation of key themes that emerged from a study of factors influencing decisions to admit to inpatient mental health facilities [taken from Nathan et al. (2)].

The consequence of the decision to admit a patient to hospital also has significant implications to the patient, the staff and the service. In a previous study, the authors investigated clinical decision-making in the context acute psychiatric admissions (2). The authors found that in addition to patient-related clinical and risk factors, clinicians were influenced by a range of non-clinical factors (**Figure 1**). Prominent among these factors were concerns about the consequences to the clinician of outcomes related to their decision-making (so-called ‘threat/fear factors’).

Whereas a key focus of clinical risk assessment in mental health settings is the possibility of harm suffered by the patient or by others as a consequence of the patients’ actions, ‘threat/fear’ factors relate instead to the possibility of harm to the clinician (2). At the core of this process is a clinical decision being made while the clinician is contemplating the patient’s future involvement in a patient safety incident. Clinical experience would suggest that as part of this future-based thinking, the clinician may be liable to anticipate not just the possible future incident itself, but also the retrospective scrutiny of their decision-making following the imagined incident (4, 14).

There is a recognition that clinicians can experience a sense of being blamed and victimized by such scrutiny processes (15). After making a decision, the clinician may discover, on learning certain outcomes, that an alternative would have been preferable (either to the clinician, patient or organization). In non-healthcare research, outcome bias and anticipated regret (or regret aversion), the counterfactual thinking of what might happen and how they might feel if they obtain a less than perfect outcome, has been shown to impact human decision making on the individual and group level (16, 17). There are no studies that have examined the nature of this type of thinking (recalled or anticipated consequences) during a decision-making process in mental health care.

Developing an understanding of the way the clinician conceptualizes future threat when they are making decisions is important for three main reasons. Firstly, this perceived threat is a potentially important influence on decision-making and thus has the potential to affect patient care and outcomes. Secondly, given that the ways in which clinicians engage in such thinking and the impact it has on their decisions is liable to vary, this may be a process that accounts for interindividual clinician differences in decision-making thresholds. Thirdly, a better understanding of this thinking pattern should inform how health systems respond to patient safety incidents (managing the need to both improve safety and clinical decision-making).

## Aims

The objective of the current study was to delineate consequences recalled and anticipated by mental health clinicians making decisions under uncertainty. The study aimed to examine the consequences clinicians considered when making decisions to admit patients to acute psychiatric inpatient care.

## Method

This study involved the additional analysis of data gathered in a study of decision-making (2). The focus of the study was the broad types of influences on clinical decision-making in an acute psychiatric scenario (e.g., assessment for an admission to hospital for an individual in crises). The data covered a range of factors that influenced decision-making where the focus group topic-guide was designed to ensure a focus on the range of reasons for their decisions. Within this data there was a focus on consequential decision-making. This additional analysis has taken a narrower frame of reference by focusing in on this one type of influence and specifically examining the nature of the thought processes linked to clinician risk. These data emerged from the focus groups and were not in direct response to targeted prompts within the topic guide but were clearly indicating a common element running through the focus group discussions that warranted more in-depth analysis.

### Participants

The types of clinicians directly involved in decisions to admit patients to acute hospital care were identified from a review of NHS mental healthcare service models and policies. Clinicians from these groups were recruited and allocated to one of four groups., namely (i) non-medical CRHTT practitioners, (ii) non-medical liaison psychiatry practitioners, (iii) Approved Mental Health Professionals (AMHP), and (iv) Consultant Psychiatrists. The CRHTT is a service that provides short-term intensive care at home for individuals at crisis. The aim of the service is to treat people at home to try to prevent hospital admission. They are often seen as the primary ‘gatekeepers’ for hospital admission from the community. Liaison practitioners were the second group and are clinical based professionals in acute physical health hospitals who assess and treat patients with mental health problems in hospital emergency departments and inpatient wards. The third group, AMHPs, were defined as a professional with a specific non-medical role in the decision-making process to admit patients to hospital under the provisions of the Mental Health Act. The final group, Consultant Psychiatrists, were involved in decisions to admit patients involuntarily and were consulted if the case was considered to be complex.

The study recruited individuals from each clinician-group from an NHS mental health trust in the North West of England, UK. The NHS trust is a large provider of community and hospital based mental healthcare.

### Design

The study was qualitative research conducted in the form of six focus groups. Each clinician group type had their own focus group, and staff were invited to participate in focus group discussions. Focus groups were used to explore staffs’ knowledge, experiences, and decision-making processes, and to examine how and why they make those decisions. Semi-structured focus group schedules were designed with questions guiding the group facilitator. This included questions related to decisions around admitting patients to hospital and was informed by a review of the literature and the study aims.

### Procedure

The focus groups were facilitated by a clinician (a senior psychiatric nurse), a clinical academic (a consultant psychiatric and academic), a service user representative, a research manager, and a university academic. The facilitator used the semi-structured focus group schedule to prompt discussions around decisions to admit patients to hospital. Participants were asked in general terms to talk about how and why they made decisions. Groups were encouraged to talk in more detail about the range of reasons for their decisions.

Written informed consent was obtained from participants. The duration of each focus group was approximately one hour. They were recorded using a digital audio recorder and transcribed verbatim.

### Patient and public involvement and engagement

PPIE was used to ensure an individual with a lived experience of being admitted to mental health services contributed to the inception of the study, the development and refinement of recruitment, data analysis and dissemination. The PPIE representative introduced the study to the clinician-groups across the trust as a method of approach.

### Data analysis

All transcripts were analyzed by authors AC and SBh. Data was analyzed following the principles of qualitative thematic analysis. The Framework Method for the management and analysis of qualitative data was used (18, 19). Familiarization and exploration of the data set as a whole identified concepts that warranted further explanation due to the high frequency of themes. The data set was assessed to identify these important themes and to evaluate how themes manifest in the data to generate new insights. An iterative coding process was used by authors AC and SBh who developed, reviewed and refined themes related to the research question. Findings were then critically discussed with author RN as an additional peer review engagement to help evaluate the interpretations and findings by introducing an alternative perspective. Final themes were identified and agreed.

Quantitative content analysis was used to investigate the proportion of recalled and anticipated consequences that were negative to self or others.

### Ethical approval

Ethical approval was obtained from the NHS Trust’s research and development department and the University of Liverpool Ethics Research Committee prior to study commencement (Reference number: 2161). All participants were informed about the study via an invitation email that provided details of the study, a participant information sheet and the consent form. Data was stored/handled in accordance with the General Data Protection Regulation rules.

## Results

There were thirty-eight participants who took part in the six focus groups between 25 June 2017 and 27 July 2017. **Table 1** shows the participants in each of the focus groups per clinician group.

**Table 1:** Participants who took part in each of the six focus group discussions.

Focus group	Clinical group	*N =* 38
1	CRHTT^*^ (1)	7
2	CRHTT (2)	6
3	CRHTT (3)	5
4	Consultant psychiatrists	6
5	AMHP^**^	10
6	Liaison psychiatry team	4

^*^Crisis resolution home treatment teams^1^.

^**^Approved mental health professionals^2^.

### Content frequency analysis

A content frequency analysis indicated that the recalled and anticipated consequences were overwhelmingly, but not exclusively, negative. In total, there were a total of 64 responses with more anticipated (*n=*41/64, 64%) than recalled (*n=*23/64, 35%) consequences within the focus groups. Of those consequences, the majority were negative (*n=*60/64, 94%). Anticipated consequences were more likely to be negative (*n=*40/41, 98%) than recalled consequences (*n*=20/23, 87%). The location of the effect of the recalled or anticipated consequences was largely directed towards the self (*n=*52/64, 81%) and of those directed to the self, the majority were negative (*n=*49/52, 94%).

### Thematic analysis

Three overarching multi-dimensional themes and seven subthemes were identified from the data (**Table 2**).

**Table 2:** Main themes and subthemes of anticipated/recalled consequences of clinical decision-making.

Theme	Subtheme
1. Location of the effect of the consequence	- Self - Other
2. Location of the origin of the consequence	- Specific - Nonspecific
3. Nature of the consequence	- Emotional correlate - Intent attribution - Valence

#### Theme 1: location of effect of consequence

##### Subtheme 1.1: to self

There was a significant focus on the effect to oneself from anticipated and recalled consequences of the clinician’s decision-making. This was overwhelmingly a negative consequence to self. A regular theme was concerns around accountability and external scrutiny about the clinician’s clinical practice:

*“they will more than likely go out and do something and then your practice is then looked upon.” (FG6)*

*“they are going to scrutinize the decision-making you’ve made and you could be subject to criticism” (FG5)*

*“I make a decision on someone, and they go and do something, and I’m held accountable for it I don’t fancy making that decision.” (FG6)*

As well as recalled or anticipated scrutiny from others from their decision-making, there was a focus on their own negative internal criticisms from their decision. The anticipated consequences were negative self-reflective practices that stems from uncertainty around likely outcomes within the original decision-making process:

*“I think you do even though you’ve made that decision you still you still analyze that decision … did I make that, did I, did I really make that right decision.” (FG2)*

*“to go home with that risk and not sleep for the next 3 or 4 nights and you know.” (FG5)*

The impact of the negative recalled and anticipated consequences on their decision-making and clinical practice was noted. A common theme was how the recalled or anticipated consequences may make them more risk averse in the future decision-making. There were no discussions about how possible outcomes may be related to or impact the system within which they work. When this was directly questioned by the focus group facilitators, the respondents denied any influence from system and situational factors:

*“you might have you know quite a high percentage of you thinking someone isn’t going to do something but that very serious thing if they did pushes you on to the more risk averse.” (FG5)*

*“once you have a bad coroner’s for whatever reason it makes you think very hard of how are you going to conduct your professional life.” (FG4)*

*“risk averse and I think almost covering all bases which makes it much slower in terms of decision making and also then you start saying no to a lot of things.” (FG4)*

##### Subtheme 1.2: to other

The anticipated and recalled consequences to others emerged less from the analysis. Often when the location of the effect was directed to others it was also linked to a consequence to self. The location of effect of the consequence was most commonly the patient coming to harm from a serious adverse event:

*“if you miss something obvious will look very silly and will not have a good outcome for the patient.” (FG4)*

*“worse case what happens if he does go out and he does harm somebody and they look at all the documentation.” (FG1).*

The decision to admit an individual to hospital was rarely, if ever, framed as a positive therapeutic decision and/or a positive risk management decision. The consequence of a hospital admission was seen negatively to the other e.g., the patient and to the organization. This was evident even when the discussion centered around what was described as evidence-based clinical care. This was also particularly apparent when the discussion was about whether to admit a patient with a diagnosis of personality disorder, where patients with features of a personality disorder diagnosis were thought to present with problems if placed in a hospital setting:

*“you do deskill people I think … it sort of confirms the fact that they can’t cope.” (FG1)*

*“and it was wholly inappropriate for her to be admitted but what else.” (FG6)*

#### Theme 2: location of origin of consequence

##### Subtheme 2.1: specific origin of consequence

A predominant finding within the analysis was a specific recalled or anticipated consequence to self of the decision being reviewed in coroner’s court, a specific origin of the consequence. The recalled or anticipated consequence of going to coroners’ court was largely negative and was associated with negative consequences to self (subtheme 1.1). The fear of their decision being scrutinized in coroners’ was thought to be a factor in the decision-making process:

*“but like the threat of even coroners’ court.” (FG1)*

*“I’m concerned about this I don’t want to be in front of the coroner.” (FG6)*

The other specific origins of the consequences were from within the organization where the clinician’s work. This was predominantly a negative recalled or anticipated consequence from management and directors within the different levels of the organization as well as the internal investigatory processes within the organization.

*“blame is lingering around at the back of your … corporate services support is as well isn’t it. “ (FG2)*

*“from upper management.” (FG1)*

*“I think people also fear … the RCA [root cause analysis] process.” (FG3)*

These discussions referenced how availability of positive support from within the healthcare system could allow clinicians to make positive risk management decisions. The feeling of being supported was linked with negative recalled consequences of previous investigatory processes following an incident.

*“what I think helps more is for teams to feel that … there are no blame culture and that if something goes wrong if they do take positive risks then there will support around, I think the standards almost goes the other way around we are saying actually we are going to come back and say not done this this this in every investigation that happens.” (FG4)*

##### Subtheme 2.2: non-specific origin of consequence

The non-specific origins of the consequences spoke of the feeling of fear from the consequences of the assessment, with a general negative perception of consistent threat/fear that may influence their decision-making.

*“on your head be it sort of thing.”(FG3)*

*“it’s hanging over all of those all the time.” (FG2)*

The focus groups also referenced the overall beliefs, values and expectations within the organization, and how that non-specific origin of the consequence can impact on their decision-making. Thus, the organizational culture increased the perception that the clinician’s decision-making would lead to negative anticipated and recalled consequences.

*“I think the culture is there is a blame culture whether we like it or not, whether we accept it or not there is I think certainly as a practitioner you feel that anyway” (FG1)*

*“yeah definitely feel there’s a sense of failure and blame. I feel it’s totally the opposite it’s a blame culture, we’ve been through it with a certain gentleman in this team and we were hounded and we had big meetings and we were told we’d failed this gentleman and the practitioner involved went through a horrendous time but as a team we were told we’d failed” (FG1)*

#### Theme 3: nature of consequence

##### Subtheme 3.1: emotional correlates

Emotional rewards and punishments were associated with the recalled and anticipated consequences of the decision-making. The emotional correlates were overwhelmingly negative, both when deciding to admit a patient and when deciding to not admit a patient. Both decisions were met with feelings of anxiety, failure and fear. The location of effect and origin of the consequence associated with the negative emotional correlates were largely to self (subtheme 1.1) and organizational factors (subtheme 2.1 and 2.2), and were found to be similar for recalled and anticipated consequences for both admitted or not admitted a patient:

*“sometimes I think you feel like you’ve failed if you’ve admitted.” (FG5)*

*“if you didn’t admit I would be anxious.” (FG5)*

The focus group attributed changes to their behaviors should they feel “*uneasy”* about a decision and related this to a negative anticipated consequence:

*“I would justify it on my documentation unduly probably with reams and reams of paperwork that probably isn’t warranted but that is only because of the potential consequence.” (FG3)*

##### Subtheme 3.2: intent attribution

The focus groups described the interpretation of others’ intentions to them associated with their recalled and anticipated consequences. The interpretation is that they would be the integral explanatory factor for the negative recalled or anticipated consequence:

*“unfortunately some people will commit suicide that’s a known fact but I think we as practitioners would feel kind of like the backlash of that if that was to happen and we’d been involved.” (FG1)*

*“and you get really criticized” (FG6)*

*“we were hounded and we had big meetings and we were told we’d failed.” (FG1)*

Within the focus groups the attribution of intent following a negative consequence was internal (i.e., to the clinician) and associated with a negative emotional correlate. The clinicians thought that the inferences generated by others about their decision-making process was critical and negative. There were discussions of how the patient’s individual decision-making and autonomy may be a causal explanation for a possible negative outcome (e.g., self-harm incident). However, the anticipated and recalled consequences from those outcomes were still attributed to the clinician making the decision about the individual’s care. The focus groups did not discuss other external (e.g., situational) factors or system factors. On exploration by the interviewer the clinicians denied that other factors may influence their decision-making.

##### Subtheme 3.3: valence

A valence-based theme emerged from the analysis, revealing a significant dimension of emotion. These were perceived negative emotions associated with recalled and anticipated consequences. A common theme was a preparedness for a worst-case scenario, highlighting a negative valence system in response to aversive situations or context.

*“in worst case scenario that obviously that you know that’s quite a traumatic thing for anybody.”(FG1)*

*“I always think oh my god you know worse case again, worse case what happens if he does go out and he does harm somebody.” (FG1)*

The perceived negative events were often seen as unpredictable, and attempts were made within the focus group discussions to link the uncertainty with risk/probability-based judgements. These appraisals relevant to the perception of risk were linked with threat/fear based emotional correlates.

*“I’m sure you know 90% of the time obviously he doesn’t do anything that’s why I’m taking that risk and you know but there’s always the 1% chance isn’t there.” (FG5)*

*“you might have you know quite a high percentage of you thinking someone isn’t going to do something but that very serious thing if they did pushes you on to the more risk averse.” (FG5)*

## Discussion

### Main findings

The decision to admit a person to an inpatient mental health facility involves inherent complexity and uncertainty. Decisions are often made with limited information and doubt over the patients’ own wishes. The clinician’s choices are rarely certain as to their potential outcomes where these professionals often make risky decisions that are likely to be influenced by wider human and system related factors. The clinician groups who make these decisions in UK clinical practice are highly heterogenous, with practitioners from different roles, services and with varying degrees of experience. This study involved focus group discussions with purposively selected groups of clinical decision-makers who routinely undertake these clinical decisions in everyday practice. The study found that clinician’s regularly recall and anticipate consequences of their decision-making. When facing uncertainty, the consequences recalled or anticipated are overwhelmingly negative and directed towards themself, rather than to the patient or components of the healthcare system.

The recalled or anticipated consequences were almost exclusively personal or imagined experiences of a negative appraisal that resulted from an actual or perceived adverse event (e.g., patient suicide). The decision-making process not only involved the formulation of clinical and risk needs, but also incorporated the clinician’s fear of the consequences. The process involved the assessment and formulation of a patient (other); however, the recalled and anticipated consequences of that decision predominantly involved the decision-maker (self). The focus groups identified that consequences were mostly organized within a negative valence system in response to aversive situations with the origin of the consequence mainly coming from external scrutiny from peers, organizational leadership, and the patient safety system/processes (safety culture, incident investigation). The threat/fear factors are likely to have implications for clinical decision-making, patient care and outcomes, patient safety responses, and professional well-being.

### Interpretation of findings

The findings show an over-arching theme of loss/threat aversion associated with the risk/probability judgements. Behavioral economics studies indicate risk/probability judgements are liable to distortion by predicted negative consequences (9, 20). Specifically, (i) attention is drawn more to negative than positive events (negativity bias); (ii) avoiding losses is preferred over making equivalent gains (loss aversion); (iii) probability judgements are biased by readily available memories (availability heuristic) which are enhanced by associated negative emotions (subtheme 3.3) (9, 21). When making decisions under uncertainty, our value-based statistical thinking is no longer sufficient and heuristic thinking is often used (22). In theory, clinicians will use evidence-based clinical reasoning through deliberative, reflective, effortful cognitions. However, sometimes, and especially when faced with uncertainty, our reasoning processes can be “hacked” by an automatic, non-conscious cognitive system (23, 24). This system relies on intuitive processes, guided by heuristics and biases that can distort our choices.

Our study demonstrates that this system may be an integral component of the decision-making process in mental health care. A systematic review of cognitive biases and heuristics in medical decision making showed that the presence of bias was prominent in most research studies, but studies relied on hypothetical scenarios, lacking the evaluation of actual real-world clinical decision-making (1). Of the included studies, only five out of the 213 (2%) studies included were performed in the mental health setting (1). Studies examining clinical decision-making have utilized individual interviews with psychiatrists showing that the decisions that are being made are dependent on the level of risk and uncertainty, where these uncontrollable factors were thought to be sources of error and bias in the decision-making process (25). Our study examined real-world decision-making with a sample of professional’s representative of actual decision-makers, identifying that the clinician conceptualizes and frames this risk and uncertainty under a fear/threat framework, which is likely to have an important impact on their decision-making.

Conflictingly, the decision to admit or not to admit were both linked with negative recalled and anticipated consequences to self. This was particularly evident in focus group discussions that centered around decisions to admit individuals whose primary diagnosis was personality disorder. This study found that admitting a patient to hospital is linked to a recalled consequence of negative emotions (e.g., sense of failure). However, not admitting a patient to hospital regularly results in negative and fearful anticipated consequences. The focus groups revealed an ideal scenario that the decision is framed against, i.e., we should not admit a patient. This may be associated with the clinician’s personal experience or linked with the policies, culture and safety needs of the organization. This prior expectation is likely to distort their perceptual representation of the anticipated consequences when faced with the decision to admit or not in real-world clinical practice. The health care system needs to be mindful of how these organizational and system-wide factors can influence day-to-day clinical decision-making.

Key decisions in clinical settings are influenced by multiple factors, including evidence-based best practice recommendations, clinician-related factors, patient related factors, and contextual environmental and system influences (2, 5, 25). The authors previous work showed that critical elements of decision-making to admit patients to hospital include threat/fear of the decision-making consequences and interpersonal dynamics (2). The additional analysis of the threat/fear element showed the importance of this factor when clinicians make decisions. This threat/fear is likely to be amplified by the inherent variability (i.e., the uncertainty) and potential seriousness of outcomes in mental health patients (e.g., suicide, homicide) (26). The epistemic uncertainty due to the poor understanding of probability and prediction with these rare events further adds to the complexity, unpredictability and emotional valence of clinical decision-making (27). With uncertain high stakes decision-making, if the health care system and organizational objectives are at odds with the complexity and fallibility of the decision-making in daily practice, risk tolerance may reduce, and risk aversion may arise (28). Clinicians incentivized to operate behind organizational goals of zero harm may distort and influence decision-making to avoid loss. Unrealistic expectations for clinical decision-making in risk assessment and management can carry a variety of significant costs to patients, professionals and clinical services (29).

Organizations need to have an awareness of how professionals working within the health care system conceptualize future threat when making decisions. An organizational objective and culture that results in anticipated/recalled consequences to avoid/reduce admission but also criticize adverse events when admission is decided against will likely impact on the clinicians making that decision. The implication of our findings for the safety system is that we need to be mindful of the perception that hindsight informed scrutiny of past/future decision making is critical and delivered with negative intent. This correlates with research evaluating the impact of complaints and scrutiny on doctors’ clinical practice, where being subject to complaint procedures significantly impacted psychological well-being and lead to more risk averse defensible practice (15, 30). Health services need to be aware of the dangers of the scrutiny felt by clinicians within healthcare organizations, especially in the NHS, UK, where workforce pressures are affecting staff retention and recruitment (31). Thought needs to be given to how incident reviews are undertaken, and that the findings and recommendations are presented to reduce the effect on the clinician.

In mental health care, intuitive practices under uncertainty are inescapable, thus being aware of these biases, system-wide influences, and the associated risks of them on our decision-making is integral for improving clinical decision-making. With an overwhelming focus on negative consequences to self, this loss aversion may result in sub-optimal healthcare choices. There may be a propensity to be risk averse and paternalistic, which may be enhanced further if clinicians do not feel supported to take reasonable risks. The self-served bias will impact on patient autonomy and choice, leading to more clinician-directed care than shared decision making (32). There may be opportunities to improve awareness of biases and heuristics amongst clinicians, and to train heuristics through experience, to refine them in a way that optimizes clinical decision-making under uncertainty. This would hopefully influence patient care and outcomes. New technologies, improvements in models to assist in risk prediction, and multi-criteria decision support interventions require further high-quality evidence to determine their role in clinical decision-making (3, 27, 33). Consideration should be given to revising guidance within mental health organizations to ensure that the admission/discharge and care pathways protocols encompass non-clinical factors and embrace uncertainty. Further research is needed to assess the impact of wider system influences and to investigate the patient’s involvement in the decision-making process and within safety incident related investigations. The involvement of patients is pertinent given patient NHS satisfaction is trending downwards (34).

### Strengths and limitations

A strength of the qualitative methodology is the number of participants (*n=38)*, the representative sample of clinician groups, and the interactions amongst participants that provided detailed descriptions of their experiences. The clinician-groups were chosen from a review of NHS service models and policy, where the consultant psychiatry group were not categorically distinct with regards to clinical role (i.e., consultant psychiatrists within this group may also work within liaison psychiatry and CRHTT). There are possible strengths and limitations from using different clinician groups. A strength is the increased likelihood of group consensus and engagement. A limitation is that pre-existing team dynamics may transpose into the focus groups and may have silenced participants with experiences conflicting to that of the team. The quieter participants may have benefited from an alternative qualitative format, i.e., 1:1 interview, as the focus group discussions may have dissuaded those with dissenting experiences, or perceptions of hierarchies within groups, from freely expressing themselves. Future research could consider if the defined roles of different clinician teams influence their decision making and whether anticipated/recalled consequences are different between psychiatrists and other clinician groups.

Another limitation is that the participants are all limited to one geographical area and one organization. This may mean that some of the data captured is specific to one organization than across mental health services more generally. Despite the detailed descriptions of clinicians recalled and anticipated consequences, this study could not determine the extent to which this influences decision-making.

## Conclusions

The results of this study provide an insight into the nature of losses predicted by clinician’s consequent upon their decisions. In day-to-day practice, healthcare professionals are not only considering patients’ clinical and risk needs, but they routinely contemplate how their decision-making will be scrutinized in the event (however unlikely) of a patient safety incident. Understanding more about the decision-making processes under uncertainty in mental healthcare can inform steps that aim reduce the associated adverse effects, to patients and professionals. A better understanding of how risk and safety is operationalized in real world complex healthcare systems reveals unintended consequences for healthcare professionals. In particular, identifying and defining non-clinical influences on clinical decision-making (such as the ‘threat/fear’ processes studied here) will inform improvements to training, service delivery, incident investigation and patient-centered care.

## Data Availability

The raw data supporting the conclusions of this article will be made available by the authors, without undue reservation.
